# Artificial intelligence in foot and ankle surgery: current concepts

**DOI:** 10.1007/s00132-023-04426-x

**Published:** 2023-08-25

**Authors:** Abhishek Vaish, Filippo Migliorini, Raju Vaishya

**Affiliations:** 1 Department of Orthopaedics and Joint Replacement Surgery, Indraprastha Apollo Hospital, Sarita Vihar, 110076 New Delhi, India; 2 Department of Orthopaedic, Trauma, and Reconstructive Surgery, RWTH University Medical Centre of Aachen, Pauwelsstraße 30, 52064 Aachen, Germany; 3 Department of Orthopaedic and Trauma Surgery, Academic Hospital of Bolzano (SABES-ASDAA), 39100 Bolzano, Italy

**Keywords:** Artificial intelligence, Foot, Ankle, Robotic surgery, Künstliche Intelligenz, Fuß, Sprunggelenk, Robotische Chirurgie

## Abstract

The twenty-first century has proven that data are the new gold. Artificial intelligence (AI) driven technologies might potentially change the clinical practice in all medical specialities, including orthopedic surgery. AI has a broad spectrum of subcomponents, including machine learning, which consists of a subdivision called deep learning. AI has the potential to increase healthcare delivery, improve indications and interventions, and minimize errors. In orthopedic surgery. AI supports the surgeon in the evaluation of radiological images, training of surgical residents, and excellent performance of machine-assisted surgery. The AI algorithms improve the administrative and management processes of hospitals and clinics, electronic healthcare databases, monitoring the outcomes, and safety controls. AI models are being developed in nearly all orthopedic subspecialties, including arthroscopy, arthroplasty, tumor, spinal and pediatric surgery. The present study discusses current applications, limitations, and future prospective of AI in foot and ankle surgery.

## Abstract (de)

Das 21. Jahrhundert hat bewiesen, dass Daten das neue Gold sind. Von künstlicher Intelligenz (KI) betriebene Technologien könnten die klinische Praxis in allen medizinischen Fachbereichen, einschließlich der orthopädischen Chirurgie, potenziell verändern. KI hat ein breites Spektrum von Teilkomponenten, darunter das maschinelle Lernen (Machine Learning), von welchem wiederum Deep Learning ein Teilbereich ist. KI hat das Potenzial, die Gesundheitsversorgung zu verbessern, Indikationen und Eingriffe zu optimieren sowie Fehler zu minimieren. KI unterstützt den Chirurgen bei der Auswertung radiologischer Bilder, bei der Ausbildung von Assistenzärzten in der Chirurgie und bei der exzellenten Durchführung maschinengestützter Operationen. Die KI-Algorithmen verbessern die Verwaltungs- und Managementprozesse von Krankenhäusern und Kliniken, elektronische Gesundheitsdatenbanken, die Überwachung der Ergebnisse und die Sicherheitskontrollen. KI-Modelle werden in fast allen orthopädischen Fachgebieten entwickelt, darunter Arthroskopie, Arthroplastik, Tumor‑, Wirbelsäulen- und Kinderchirurgie. Die vorliegende Studie erörtert aktuelle Anwendungen, Grenzen und Zukunftsaussichten der KI in der Fuß- und Sprunggelenkchirurgie.

## Introduction

The twenty-first century has proven that data is the new gold. Artificial intelligence (AI) driven technologies might potentially change the clinical practice in all medical specialities, including orthopedic surgery. AI has a broad spectrum of subcomponents, including machine learning (ML), which consists of a subdivision called deep learning (DL). AI has the potential to increase healthcare delivery, improve indications and interventions, and minimize errors. AI is the intelligence demonstrated by the machines such as computers. It has several abilities such as a) to learn, b) to reason, c) to generalize, and d) to infer meaning. AI technology adapts and integrates several problem-solving techniques such as search and mathematical optimization, formal logic, artificial neural networks, and methods based on statistics, probability, and economics. Presently, highly mathematical and statistical ML has dominated AI. These solve many challenging problems for academia. AI is the process of human-like intelligence simulated by using computer-controlled machines. It includes information, reasoning, and self-correction capability. AI is used with intelligent robots and the associated machinery to perform orthopedic surgery more accurately. These systems can detect mistakes in the given environment and provide actionable information regarding heat, light, movement, temperature, sound, and pressure and thus minimize human errors. In orthopedic surgery, AI supports the surgeon in the evaluation of radiological images, training of surgical residents and excellent performance of machine-assisted surgery. The AI algorithms improve the administrative and management processes of hospitals and clinics, electronic healthcare databases, monitoring the outcomes, and safety controls [1].

AI models are being developed in nearly all orthopedic subspecialties, including arthroscopy, arthroplasty, tumor, spinal and pediatric surgery. Klemt et al. developed and validated ML models to predict the risk of early revision following primary total hip arthroplasty (THA) [2]. Jo et al. proposed an ML model to predict the risk of blood transfusion following primary total knee arthroplasty (TKA) [3]. Merali et al. developed and validated a DL model for detecting cervical spinal cord compression in magnetic resonance imaging (MRI) scans [4]. Kunze et al. trained and tested several ML models for predicting patients who would achieve the minimal clinically important difference (MCID) in the hip outcome score-sports subscale (HOS-SS) following hip arthroscopy for femoroacetabular impingement syndrome [5]. Xu et al. developed a DL-assisted system for automated measurements and classifications pertinent to developmental dysplasia of the hip directly from plain pelvic radiographs [6].

Technological advances are happening at an accelerated speed and are being incorporated into healthcare. Several such technologies have made their way into orthopedics, such as computer navigation, robot-assisted arthroplasty and 3‑D planning. With the advent of progressive understanding and refinement of software algorithms, orthopedic surgery is now delving into AI systems. The present generations of AI algorithms help in image recognition and multivariate risk analysis, and outcome prediction. It is becoming obvious that AI and ML are likely to significantly impact clinical orthopedic practices in the short term, and will find newer applications, increased utility and the use of ML in clinical practice. AI is expected to provide solutions to the traditionally redundant and repetitive tasks that are lower on the intellectual spectrum and contribute to surgeons’ burnout and mistakes; however, AI faces several challenges including ethical deployment, regulatory issues, and its clinical superiority over traditional statistics and decision making. Several clinical applications of AI in orthopedics include the measurement of bone dimensions, and management of fractures, spinal problems and arthroplasty. It is an innovative way of using the available information to efficiently perform complex cases. This technology supports the orthopedic surgeon in the appropriate selection of surgical implants. It is a promising technology to improve the outcomes of orthopedic surgery.

The present review discusses the role of AI in foot and ankle surgery, focusing on cost implications, potential limitations and future perspectives.

## Role of AI in foot and ankle surgery

### Diagnostics

With many patients seeing nonorthopedic care providers for foot and ankle radiograph interpretation, DL and AI can be important in getting patients accurately and quickly diagnosed and referred to more specialized providers. Convoluted neural networks (CNNs), a form of DL, recognize visual patterns from raw image pixels which makes them potentially useful for medical imaging. While CNNs developed for radiographic images demonstrate high fracture detection, they are ultimately limited in that radiographs provide only a 2D representation of 3D joints. To address this, AI for ankle and foot fracture detection expands beyond radiographs to computed tomography (CT) imaging as well. Through de novo and pretrained CNNs, DL has been found to successfully detect and accurately classify 92–98% of Sanders calcaneal fracture types [7].

### Robotic applications

The field of robotics for use in surgery is not limited to robotic arm applications for intraoperative assistance. AI has been also advocated for imaging analysis, patient-specific instrumentation in preoperative planning, and robotics-aided rehabilitation [8–10].

### Management of fractures

Several authors have described the use of AI in the diagnosis and treatment of ankle and foot fractures. Ashkani-Esfahani et al. internally validated two deep convolutional neural networks (DCNN) for identifying ankle fractures from radiographs and achieved a near-perfect area under the curve (AUC) of 0.99 [11]. Kitamura et al. internally validated 5 separate CNNs for detecting ankle fractures from plain radiographs and achieved a fair fracture detection accuracy of 81% [12]. Prijs et al. internally and externally validated a DL model for detecting, classifying, and localizing ankle fractures from plain radiographs and achieved an excellent AUC of 0.92 and an accuracy of 99 % on external validation [13]. Guermazi et al. internally validated a DL model for detecting fractures from foot and ankle plain radiographs, which performed excellently with an AUC of 0.97, sensitivity per patient of 93%, and specificity per patient of 93% [14]. Olczak et al. internally validated neural network models for classifying ankle fractures from radiographs according to the AO Foundation/Orthopaedic Trauma Association (AO/OTA) 2018 classification, which showed fair to excellent performance with AUCs ranging from 0.79 to 0.99 in classifying AO types [15]. Pinto Dos Santos et al. internally validated a CNN for detecting fractures in anteroposterior ankle radiographs, which performed well with an AUC of 0.85 [16]. Ashkani-Esfahani et al. internally validated 2 DCNN models for detecting Lisfranc instability from single-view (anteroposterior) and 3‑view radiographs (anteroposterior, lateral, oblique), which performed excellently with AUCs ranging from 0.925 to 0.994 [11]. Aghnia Farda et al. internally validated a CNN model for classifying calcaneal fractures on CT images into the Sanders system, which performed well with a classification accuracy of nearly 72% after augmenting the data 17. Pranata et al. internally validated 2 separate DCNN models for detecting the presence or absence of calcaneal fractures on CT images and achieved an excellent accuracy of 98% [17]. Hendrickx et al. internally validated 4 ML and DL models for predicting patients with tibial shaft fractures and associated occult posterior malleolar fractures. The models performed well with AUCs ranging from 0.81 to 0.89 [18]. Oosterhoff et al. internally validated 5 models for predicting posterior malleolar involvement in distal tibial shaft fractures using the same data set as that in the previously described study by Hendrickx et al [19]. Oosterhoff et al. found that all the models performed well with AUCs  0.80 (highest 0.89) and 4 of 5 having a Brier score of 0.11 [19].

### Tendinopathies

Wang et al. internally validated several radiomics-based ML models for diagnosing Achilles tendinopathy from ultrasonographic images in skiers and achieved an excellent AUC of 0.99, 90% sensitivity, and 100% specificity [20]. Kapiński et al. internally validated several DL models to classify Achilles tendons injuries on MRI scans, achieving a maximum accuracy of 97.6%, a sensitivity of 98.3%, and a specificity of 99.45% [21]. Merrill et al. internally validated a logistic regression and gradient boosting model for predicting short-term complications, including mortality and readmissions, in patients who have undergone open reduction and internal fixation (ORIF) in acute ankle fractures. Both models performed similarly, with AUCs for gradient boosting ranging from 0.6979 to 0.7580 and AUCs for logistic regression ranging from 0.7101 to 0.7583 [22].

### Hallux valgus

Li et al. aimed to internally validate a DL model to detect 18 anatomical landmarks from weight-bearing radiographs, including the hallux valgus angle (HVA), hallux interphalangeal angle (HIA), first-second intermetatarsal angle (IMA), and distal metatarsal articular angle (DMAA). The observed (manual by a radiologist) and predicted (model) values of the 4 angles correlated well (intraclass correlation: 0.89–0.96, r = 0.81–0.97) [23]. Day et al. aimed to assess the performance of an AI-based software that automatically measures the M1–M2 IMA from weight-bearing cone beam computed tomography (WBCT) scans in patients with hallux valgus. The AI-based software was faster than manual measurements, correlated well with manual measurements, and had higher and nearly perfect test-retest reliability (0.99 intrasoftware intraclass correlation coefficient for both 3D and 2D IMA) [24]. Wang et al. validated a support vector machine model to classify patients with symptomatic hallux valgus using HVA, IMA, and DMAA, with a fair accuracy of 76.4% [20].

### Stress fractures

Wang et al. internally and externally tested a DL system for detecting and grading fatigue fractures (a type of stress fracture) from plain radiographs, which performed excellent (AUC 0.911, sensitivity 90.8%) in the detection of fatigue fractures for the foot images and good (AUC 0.877, sensitivity 85.5%) for the tibiofibula images. External validity for grading of fatigue fractures was not demonstrated as the DL system performed poorly with an overall accuracy of 62.9% for the tibiofibular images and an accuracy of 61.1% for the foot images [20].

### Sports injury

Diniz et al. internally validated one ML model for predicting whether soccer players would return to similar performances after Achilles tendon rupture, achieving a good AUC of 0.81 and a Brier score loss of 0.12 [25]. Lu et al. internally validated many ML models for predicting the occurrence of a lower extremity muscle strain (quadriceps, calf, hamstring, groin) in elite basketball players [26]. Among them, the XGBoost model achieved the highest AUC of 0.840, representing the best-performing model if the Brier score and calibration were also considered [25]. Jauhiainen et al. internally validated 2 ML models for predicting moderate and severe knee and ankle injuries in young basketball and floorball players (age ≤ 21 years), which performed poorly with an AUC of 0.63 for the random forest model and 0.65 for the logistic regression model [27]. Ruiz-Pérez et al. internally validated many ML models to predict lower extremity non-contact soft tissue injury in professional futsal players, which generally performed fairly, with the best model achieving an AUC of 0.767, a sensitivity of 85.1%, and a specificity of 62.1% [28]. Suda et al. internally validated several support vector machine models for classifying running experience levels based on foot-ankle kinematic and kinetic patterns to potentially assist with running rehabilitation and training. The models performed well with classification accuracies of 88.5% for less experienced runners, 87.2% for moderately experienced runners, and 84.6% for experienced runners [29].

### Plantar fasciitis

Yin et al. internally validated a neural network model for predicting patients that would achieve the minimum clinically successful therapy (decrease in the visual analogue score, VAS, by 60% or more from baseline) at 6 months following extracorporeal shock wave therapy (ESWT) in patients with chronic plantar fasciitis. The model performed well, with an overall accuracy of 92.5%, a sensitivity of 95.0%, and a specificity of 90.0% [30]. Keijsers et al. internally validated a neural network model for differentiating patients who have forefoot pain and those that do not use plantar pressure data, which performed satisfactorily with an accuracy of 70.4% [31]. Zhu et al. investigated whether AI-assisted ultrasonography-guided needle knife therapy improves the outcomes of patients treated for chronic plantar fasciitis. Patients who were allocated to the AI-assisted group evidenced statistically significant higher American Orthopaedic Foot & Ankle Society (AOFAS), lower plantar fascia elasticity scores and plantar fascia thickness at 2, 4, and 8 weeks of follow-up [32].

### Ankle arthroplasty

Hernigou et al. applied AI and ML to assist in conducting their study for developing a method of defining the ideal and patient-specific motion axes of the tibiotalar joint, intending to improve robotic-assisted total ankle arthroplasty (TAA) [33].

### Gait abnormalities

Ardhianto et al. applied DL to help with the automated measurement of the foot progression angle (FPA) from plantar pressure images, helping clinicians in assessing gait abnormalities [34].

### Miscellaneous applications

Pakhomov et al. applied ML to automate the identification and classification of foot examination findings from clinical notes as normal, abnormal, or not assessed, and their models performed well with overall accuracies ranging from 81% to 87% [35].

## Limitations

Spending billions of dollars on AI technologies, humans are still dealing with the hype of AI and have relatively failed to realize the real uses of this technology and utilize it in the most cost-effective pathway. The value of AI-based solutions should be investigated on several factors, such as ethics, value propositions, the indications of developing the algorithm, safety and risks, potential users, generalizability, quality and validity, and the current limitations to the clinical translation. One of the limitations of using AI was that the images from a single institution will have identical slice thickness and pixel dimension. As other institutions have different imaging technology and image dimensions, the development of deep learning models that have been trained with diverse imaging pools and that can accommodate differences in source imaging is essential.

## Future implications

### Clinical implications

While foot and ankle surgery has lagged behind other orthopaedic specialities, employing and studying robotics more extensively in this field is necessary. CNNs can be trained for autonomous outcome prediction and are currently focused on fracture detection with projected optimization in a multitude of clinical settings. Lastly, considering post-injury and post-surgey outcomes, robotic foot braces, emulators, and assistive limb devices have a variety of adaptive functions with options for real-time patient feedback that profoundly individualize patient rehabilitation.

### Total ankle arthroplasty

Advancements in robotic-assisted TKA and THA demonstrated good clinical outcomes, showing a promising future for application in TAA; however, because of the broad range of foot and ankle surgery with lower volumes in singular procedures than arthroplasty, significant cost barriers exist for the widespread adoption of these technologies. Translational cadaveric studies might help clarify the native mechanical strains and injury biomechanics of ankle joints, test the current TAA systems, and introduce novel machinery for hands-off fracture reduction. At the clinical end, robotics and computer-based systems are being employed for increased precision in TAA and trauma, but these developments are less extensive when contrasted with THA and TKA robotics. Therefore, contained air solutions (CAS) and robots with open technological capacities will likely be more widely adopted in the coming years for use in the foot and ankle; however, improving implant positioning with robotic-assisted TAA can lead to a reduction in long-term healthcare costs, especially given the high failure rates of TAA compared to other joint replacements. If open robotic systems are also developed with capabilities for other procedures that often accompany TAA, such as soft tissue manipulations, longitudinal costs and outcomes will likely be significantly improved both in the operative suite and for patient quality of life.

### Prosthetics and orthotics

With future improvements in ankle prostheses, orthotics, and therapeutics on the horizon, further work would help optimize the design of these systems to create more lightweight devices to reduce mechanical work on behalf of the user and to recreate better natural motion [36]. The expansion of the ankle orthosis to a foot-ankle-knee orthosis for more debilitating pathologies has also been described in the literature [37]. Other suggestions include individualized protocols that are tailored to individual patient needs rather than a standardized, one size- fits all protocol. Ultimately, patients will benefit from these technologies through modifiable products promoting individualized recovery, lending to improved post-surgery outcomes.

### Healthcare management

Advancements in AI and DL will allow for incorporation in the primary care and acute care settings for increased efficiency and accuracy of ankle pathology diagnoses. Especially regarding scenarios in which practitioners are less familiar with complex orthopedic injuries, these systems can close a gap in knowledge in practice while decreasing the cost of care and time spent interpreting radiographic imaging for more swift referrals, treatment plans, and time to surgical intervention. Given the impressive precision and accuracy of these algorithms, another application is telehealth, allowing for remote diagnostics, potentially without a radiologist’s interpretation. Beyond fracture detection, AI systems can also be employed to inform surgeons of patient-specific projected outcomes based on prior data patterns, answering questions such as “What is my patient’s risk of reoperation or implant failure?” or “How long until this patient is back to work?”.

### Research

There exists a vast potential for the application of robotics in the realms of preclinical and translational research, clinical evaluation (e.g., with AI), preoperative planning, and CAS robotics, among others. Future research should be aimed at incorporating robotic technologies specifically into surgical procedures and clinical practice, for which cadaveric translational studies have proven to be an accurate and replicable pipeline.

In vitro and in vivo gait simulators can begin to transition to human subjects; however, less invasive versions should be first developed. Additionally, because most cadaveric models in the past have been static with one plane of motion, employing more dynamic robotic simulators with more degrees of freedom will allow for a more realistic positioning of the specimens to represent biological motion better. Moreover, these static simulators apply only one or two dimensions of action, such as torque or axial load, over fixed ranges of motion. With knowledge of the complexity of joint loading and strain, it would be of interest to apply these concepts to robotic systems to mirror joint kinematics during daily activities such as walking, lunging, and pivoting. This would also necessitate quantification of these types of loads during these activities, which has yet to be elucidated. This research will enrich our understanding of the ankle joint, which can be directly applied to surgical planning and postoperative therapy and return to motion.

## Cost implications

Currently, the companies providing AI software are charging hefty fees. This is mainly because of the money which goes into research. This field is at present constantly evolving and once it is streamlined, the cost is bound to come down. Also, integration into the system of healthcare globally will increase the volume of data as well as the users. This in turn will attract multiple companies to offer this technology at a much more competitive rate.

## Conclusion

AI is spreading in foot and ankle surgery, but most models lack external validation. Currently, the majority of the models are being used for image interpretation and are performing excellently in doing so, but model performance is not robust for clinical predictions. More subject areas need to be explored in foot and ankle surgery, and models with better performance and external validation are required. The materials and methods should be described with sufficient detail to allow others to replicate and build on the published results. Please note that the publication of your manuscript implies that you must make all materials, data, computer code, and protocols associated with the publication available to readers. Please disclose at the submission stage any restrictions on the availability of materials or information. New methods and protocols should be described in detail while well-established methods can be briefly described and appropriately cited.

## Abbreviations

AI: Artificial intelligence
AOFAS: American Orthopaedic Foot & Ankle Society
AO/OTA: AO Foundation/Orthopaedic Trauma Association
AUC: Area under curve
CNN: Convoluted neural network
CT: Computer tomography
DCNN: Deep convolutional neural network
DL: Deep learning
DMAA: Distal metatarsal articular angle
ESWT: Extracorporeal shock wave therapy
FPA: Foot progression angle
HIA: Hallux interphalangeal angle
HOS-SS: Hip outcome score-sports subscale
HVA: Hallux valgus angle
IMA: Intermetatarsal angle
MCID: Minimal clinically important difference
ML: Machine learning
MRI: Magnetic resonance imaging
TAA: Total ankle arthroplasty
THA: Total hip arthroplasty
TKA: Total knee arthroplasty
VAS: Visual analogue score
WBCT: Weight-bearing cone beam computed tomography

## Declarations

For this article no studies with human participants or animals were performed by any of the authors. All studies mentioned were in accordance with the ethical standards indicated in each case.
